# Goshajinkigan, a Traditional Japanese Medicine, Suppresses Voltage-Gated Sodium Channel Nav1.4 Currents in C2C12 Cells

**DOI:** 10.1089/biores.2019.0034

**Published:** 2020-04-27

**Authors:** Ryota Imai, Shoichiro Horita, Yuko Ono, Keisuke Hagihara, Masaru Shimizu, Yuko Maejima, Kenju Shimomura

**Affiliations:** ^1^ Department of Bioregulation and Pharmacological Medicine, Fukushima Medical University School of Medicine, Fukushima, Japan.; ^2^ Department of Disaster and Emergency Medicine, Graduate School of Medicine, Kobe University, Kobe, Japan.; ^3^ Department of Kampo Medicine, Osaka University Graduate School of Medicine, Osaka, Japan.; ^4^ Department of Neurology, Matsumura General Hospital, Fukushima, Japan.

**Keywords:** C2C12, goshajinkigan, Kampo, skeletal muscle, traditional Japanese medicine, voltage-gated sodium channel

## Abstract

Goshajinkigan (GJG) is a traditional Japanese Kampo medicine used clinically to treat muscle pain in Japan. However, its underlying mechanism remains unclear. Since voltage-gated sodium channel (Nav) 1.4 is involved in skeletal muscle contraction, we investigated the possibility that GJG may affect Nav1.4 currents. By using an electrophysiological technique on skeletal muscle cell line C2C12, we found that GJG suppresses Nav1.4 currents in C2C12 cells. It is suggested that GJG may improve skeletal muscle stiffness or cramps by inhibiting abnormal Nav1.4 excitation. GJG may act as a Nav1.4 blocker and may be useful to treat muscle stiffness and clamps as well as easing the pain.

## Introduction

Voltage-gated sodium channels (Nav) are important to initiate and propagate action potential in the excitable tissues such as the skeletal muscle, cardiac muscle, and nerve. Nine subtypes (Nav1.1–Nav1.9) have been identified in mammals with Nav1.4 predominantly expressed in the skeletal muscle.^1^ Nav1.4 is activated by sensing a motor endplate potential change, which eventually causes myofiber excitation.^2^ It is assumed that the excessive activation of Nav1.4 may cause abnormal skeletal muscle contraction. Sodium channel blockers are known to be effective for muscle stiffness in patients with a mutation in the *SCN4A* gene encoding Nav1.4 and for muscle cramps in amyotrophic lateral sclerosis (ALS) patients.^3,4^

Goshajinkigan (GJG), a traditional Japanese Kampo medicine, has been used clinically in Japan to treat lower back pain, rhigosis or numbness of the extremities, and melosalgia. GJG is also reported to improve chemotherapy-induced and diabetic neuropathy.^5,6^ However, recently, the effects of GJG on the skeletal muscle have been focused.^7–9^ Some clinical reports show that GJG relieves pain caused by skeletal muscle cramps.^10–12^ Therefore, GJG may suppress abnormal skeletal muscle excitation and relieve muscle stiffness or cramps.

To date, no reports show the effects of GJG on the skeletal muscle excitability. In this study, we investigated whether GJG can suppress Nav currents that regulate muscle excitability of C2C12 cells.

## Materials and Methods

### Cell culture

Cell culture was performed according to the previous report.^13^ Murine C2C12 myoblasts (RIKEN Cell Bank, Ibaraki, Japan) were cultured in high-glucose Dulbecco's modified Eagle's medium (DMEM) supplemented with 10% (v/v) fetal bovine serum, 100 U/mL penicillin, and 100 μg/mL streptomycin at 37°C in a humidified atmosphere containing 5% CO_2_. After the cells reached confluence, the culture medium was changed to high-glucose DMEM supplemented with 2% (v/v) heat-inactivated horse serum, 100 U/mL penicillin, and 100 μg/mL streptomycin to induce myogenic differentiation. C2C12 cells on day 5–13 after differentiation were used in experiments.

### Reagents

GJG was obtained from Tsumura and Co. (Tokyo, Japan), manufactured by spray-drying a hot water extract of a mixture of 10 crude drugs: *Rehmanniae* radix (5 g), *Achyranthis* radix (3 g), *Corni* fructus (3 g), *Moutan* cortex (3 g), *Alismatics* rhizome (3 g), *Dioscoreae* rhizome (3 g), *Plantaginis* semen (3 g), *Hoelen* (3 g), processed *Aconiti* tuber (1 g), and *Cinnamomi* cortex (1 g). GJG (50, 100, 500 μg/mL) was suspended in an extracellular solution, then centrifuged (5000 rpm, ∼3000 × *g*) for 10 min and used after filtering (0.22 μm). Tetrodotoxin (TTX, 100 nM) was dissolved in an extracellular solution.

### Electrophysiology

Whole-cell patch-clamp recordings were performed referring to the previous report.^14^ Spindle cells were selected for the electrophysiological experiments. All recordings were performed in voltage-clamp mode at room temperature (22–25°C) using an EPC-800 patch-clamp amplifier (HEKA Electronics, Lambrecht/Pfalz, Germany) filtered at 1 kHz. Data were digitized with an analog-to-digital converter (Molecular Devices, CA) and stored on a computer using Clampex 10.5 software (Molecular Devices). Patch electrodes (3–6 MΩ) were filled with an internal solution containing (in mM) 105 CsF, 10 NaCl, 10 ethylene glycol tetraacetic acid, and 10 2-[4-(2-hydroxyethyl)-1-piperazinyl]ethanesulfonic acid (HEPES) (pH 7.3 with CsOH). The extracellular solution contained (in mM) 140 NaCl, 2.5 KCl, 1 MgCl_2_, 1 CaCl_2_, and 10 HEPES (pH 7.4 with NaOH). Data were analyzed using Clampfit 10.5 software (Molecular Devices). For the measurement of Nav currents, the cells were held at −120 mV and the currents were evoked by 20 msec voltage depolarization to voltage values between −80 and +50 mV in 10 mV increments. GJG or TTX was applied to the cells through bath perfusion. The currents were recorded 3–10 min after exposure to these drugs or washout. GJG concentration–response curve of Nav was fitted with the following equation: G/Gc = *a* + (1 – *a*)/(1 + ([GJG]/IC_50_)^*h*^), where [GJG] is the GJG concentration, *a* is the fraction of unblocked current at saturating [GJG], IC_50_ is the [GJG] at which inhibition is half-maximal, and *h* is the slope factor (Hill coefficient). To control for possible rundown, Gc was taken as the mean of the conductance in control solution before and after GJG application. The mean ± standard error of the mean (SEM) values of IC_50_ and *h* were calculated by fitting each individual dose–response curve and calculating the mean of the values obtained.

### Quantitative reverse transcription-polymerase chain reaction

Total RNA from C2C12 cells after differentiation induction [by replacing 10% (v/v) fetal bovine serum with 2% (v/v) horse serum] was extracted using ISOGEN (Nippon Gene, Tokyo, Japan) according to the manufacturer's instructions, which was then followed by quantitative reverse transcriptase-polymerase chain reaction (qRT-PCR) assay. In brief, total amount of 1 μg RNA was used for reverse transcription, and the casein kinase 2a2 (csnk2a2) gene was used for normalizing gene expression. The primers used for csnk2a2 and Nav1.4 genes are 5′-GGAGGCCCTAGATCTTCTTG-3′ (forward)/5′-CGCGTTAAGACGTTTTGATT-3′ (reverse), and 5′-GCCTTGCGCTCTCTGACTTG-3′ (forward)/5′-ACAGCGTGGGTGACACAAAGTA-3′ (reverse), respectively.

### Statistical analysis

The statistical analyses were performed by two-way repeated-measures ANOVA with *post hoc* Bonferroni's test for current–voltage relationship curves. All data were expressed as mean ± SEM.

## Results

**FIG. 1.**
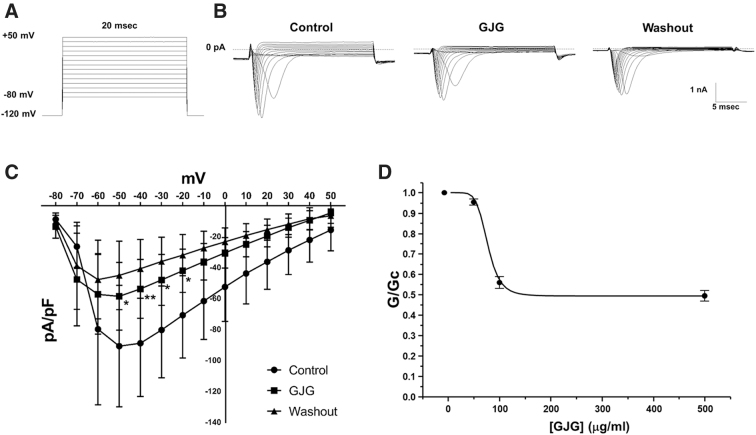
The effects of goshajinkigan (GJG) on Nav currents of C2C12 cells. **(A)** A voltage protocol for evoking Nav currents. **(B)** The representative Nav currents in control solution, GJG solution (500 μg/mL) and after the washout. **(C)** Current–voltage relationship curves in control solution, GJG solution (500 μg/mL), and after the washout (*n* = 5, the control group was compared with the GJG group and the washout group by two-way repeated-measures ANOVA *post hoc* Bonferroni's test, *p < 0.05, **p < 0.01, control vs. GJG). **(D)** Mean relationship between GJG concentration and Nav conductance (G), expressed relative to the conductance in the absence of GJG (Gc) for Nav (*n* = 5). The smooth curves are the best fit of the equation to the mean data, with the following parameters: IC_50_ = 73.13 μg/mL, *h* = 6.09, *a* = 0.49. ANOVA, analysis of variance; GJG, goshajinkigan; Nav, voltage-gated sodium channel.

To investigate whether GJG can suppress Nav currents in C2C12 cells, we measured Nav currents before and after the application of GJG using the whole-cell patch-clamp technique. Figure 1A shows a voltage protocol for recording Nav currents. Figure 1B shows representative Nav currents in control solution, GJG solution, and after the washout. Current–voltage relationship curves indicate that GJG significantly suppressed inward Nav currents between −50 and −20 mV compared with the control group, and that the suppressed currents were not recovered by washout (within 10 min) (Fig. 1C). The amplitude of current varied depending on the recorded cells. This may be due to the difference in expression rate in each cell. The inhibition by GJG on the peak current in −50 mV was shown at dose dependency (IC_50_ = 73.13 μg/mL) (Fig. 1D). However, Nav current was incompletely inhibited at even the highest concentration.

**FIG. 2.**
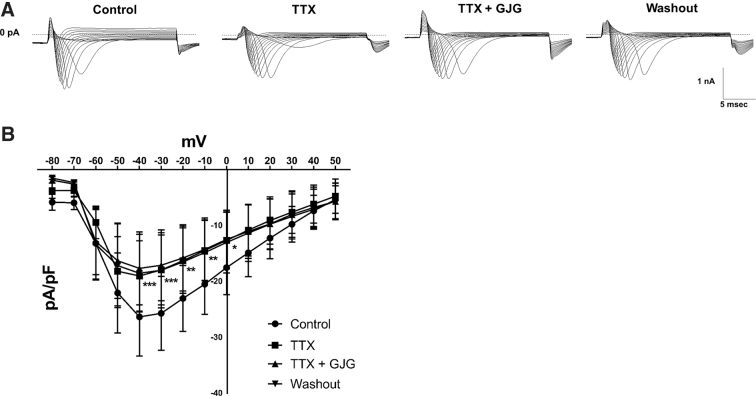
The effects of goshajinkigan (GJG, 500 μg/mL) or tetrodotoxin (TTX, 100 nM) on Nav currents of C2C12 cells. **(A)** The representative Nav currents in control solution, GJG solution, TTX+GJG solution, and after the washout. **(B)** Current–voltage relationship curves in control solution, TTX solution, TTX+GJG solution, and after the washout (*n* = 6, the TTX group was compared with the control group, the GJG+TTX group and the washout group by two-way repeated-measures ANOVA *post hoc* Bonferroni's test, *p < 0.05, **p < 0.01, ***p < 0.001, control vs. TTX). TTX, tetrodotoxin.

We sought to clarify the subtypes of Nav blocked by GJG. Since C2C12 cells are reported to express Nav1.4 and Nav1.5,^15^ we compared the expression level of the genes in our C2C12 cells by qRT-PCR. The result showed that the expression level of Nav1.4 is larger than that of Nav1.5 (data not shown), suggesting that Nav1.4 may contribute to overall Nav current. In addition, our qRT-PCR assay confirmed the expression ratio of Nav1.4 compared with housekeeping gene csnk2a2 was ∼27.6. We distinguished Nav1.4 (TTX-sensitive) currents from Nav1.5 (TTX-resistant) currents by applying 100 nM TTX^1,16^ and investigated whether GJG can affect the remaining non-Nav1.4 currents after TTX application. Figure 2A shows representative Nav currents in control solution, TTX solution, TTX+GJG solution, and after the washout. Current–voltage relationship curves indicate that TTX significantly suppressed inward Nav currents between −40 and ±0 mV compared with the control group (Fig. 2B). However, there were no significant differences between the effects of TTX alone and TTX+GJG, indicating that additional GJG application had no effect on the remaining currents after the TTX application. The currents after the TTX and GJG application were not recovered by washout (within 10 min). These results suggest that GJG may suppress Nav1.4 but not Nav1.5 in C2C12 cells.

## Discussion

Various natural ingredients are known to block Nav.^17^ Since GJG is composed of 10 crude drugs, GJG may contain ingredients that are capable of blocking Nav. Indeed, some ingredients binding to Nav have been identified in GJG, especially in processed *Aconiti* tuber.^18,19^ Moreover, it has been reported that lappaconitine or its metabolite *N*-deacetyllappaconitine, ingredients of processed *Aconiti* tuber of GJG, can block Na current of the trigeminal neurons, cardiac muscle cells, or hippocampal pyramidal neurons.^20^ However, Nav1.4 is not expressed in these tissues. To our knowledge, the inhibitory effects of the ingredients of GJG on Nav1.4 have never been studied. Thus, this study may provide a new insight that the ingredients of GJG can block Nav1.4 in the skeletal muscle.

Although, as mentioned earlier, lappaconitine is reported to block Na current in cardiomyocytes (Nav1.5),^20^ our results showed that GJG may not affect Nav1.5 in C2C12 cells. The reason why GJG failed to inhibit Nav1.5 currents is unclear, but it may be due to the interaction between various ingredients in GJG^18,19^ or the difference of the tissue used in experiments. Further studies for the effects of GJG on Nav1.5 and the effects of each ingredient on Nav current are required.

Some analgesic mechanisms of GJG for neuropathic pain have been reported.^21^ It is assumed that the analgesic effects of GJG are mainly exerted by functional modification in the central nervous system.^22^ However, the previous reports suggest that GJG may decrease cold hypersensitivity through peripheral TRPA1 and TRPM8 inhibition, and that lappaconitine, 6-benzoylheteratisine, 1-benzoylnapelline, and 14-benzoyltalatisamine, ingredients of processed *Aconiti* tuber, may exert analgesic effects through the peripheral Nav inhibition.^18,19,23^ Therefore, it is suggested that GJG ingredients may reach and affect the peripheral tissue, including skeletal muscle.

Antiarrhythmic medicines such as mexiletine, which inhibit inward Na current, are effective for treating patients with the Nav1.4 gene mutation and ALS patients to prevent skeletal muscle stiffness and cramps.^3,4^ However, antiarrhythmic medicines often show adverse effects and insufficient efficacy.^24^ Therefore, we suggest that GJG may be useful as a complementary medicine for muscle stiffness and cramps caused by Nav1.4 dysfunction.

## Conclusion

Although further clinical studies are required, we suggest that GJG may become a therapeutic medicine for the skeletal muscle stiffness or cramps through blocking Nav1.4.

## Abbreviations Used

ALS: amyotrophic lateral sclerosis
csnk2a2: casein kinase 2a2
DMEM: Dulbecco's modified Eagle's medium
GJG: goshajinkigan
Nav: voltage-gated sodium channel
qRT-PCR: quantitative reverse transcriptase polymerase chain reaction
TTX: tetrodotoxin
